# Expression of human carcinoembryonic antigen‐related cell adhesion molecule 6 and alveolar progenitor cells in normal and injured lungs of transgenic mice

**DOI:** 10.14814/phy2.12657

**Published:** 2015-12-23

**Authors:** Shin‐e Lin, Anne Marie Barrette, Cheryl Chapin, Linda W. Gonzales, Robert F. Gonzalez, Leland G. Dobbs, Philip L. Ballard

**Affiliations:** ^1^ Department of PediatricsUniversity of California San FranciscoSan FranciscoCalifornia; ^2^ Department of PediatricsChildren's Hospital of PhiladelphiaPhiladelphiaPennsylvania; ^3^ Cardiovascular Research InstituteUniversity of California San FranciscoSan FranciscoCalifornia

**Keywords:** Alveolar epithelium, CEACAM6, lung injury, stem cells

## Abstract

Carcinoembryonic antigen‐related cell adhesion molecule 6 (CEACAM6) is expressed in the epithelium of various primate tissues, including lung airway and alveoli. In human lung, CEACAM6 is developmentally and hormonally regulated, protects surfactant function, has anti‐apoptotic activity and is dysregulated in cancers. We hypothesized that alveolar CEACAM6 expression increases in lung injury and promotes cell proliferation during repair. Studies were performed in CEABAC transgenic mice‐containing human CEACAM genes. The level of CEACAM6 in adult CEABAC lung was comparable to that in human infants; expression occurred in epithelium of airways and of some alveoli but rarely co‐localized with markers of type I or type II cells. Ten days after bleomycin instillation, both the number of CEACAM6^+^ cells and immunostaining intensity were elevated in injured lung areas, and there was increased co‐localization with type I and II cell markers. To specifically address type II cells, we crossed CEABAC mice with animals expressing EGFP driven by the SP‐C promoter. After bleomycin injury, partially flattened, elongated epithelial cells were observed that expressed type I cell markers and were primarily either EGFP ^+^ or CEACAM6^+^. In cell cycle studies, mitosis was greater in CEACAM6^+^ non‐type II cells versus CEACAM6^+^/EGFP ^+^ cells. CEACAM6 epithelial expression was also increased after hyperoxic exposure and LPS instillation, suggesting a generalized response to acute lung injuries. We conclude that CEACAM6 expression is comparable in human lung and the CEABAC mouse. CEACAM6 in this model appears to be a marker of a progenitor cell population that contributes to alveolar epithelial cell replenishment after lung injury.

## Introduction

CEACAM6 (also called NCA, NCA‐50/90, and CD66c) is a member of the carcinoembryonic antigen (CEA) gene family, consisting of 29 related genes, and it is expressed in apical membranes of polarized epithelial cells of many tissues where it functions as an intercellular adhesion molecule with signaling properties (Hammarstrom 1999; Kuroki et al. 2001). CEACAM6 also binds a variety of gram‐negative bacteria and mediates internalization and phagocytosis, participating in innate immune defense in the intestine (Chen et al. 1997). Over‐expression of CEACAM6 occurs in Crohn's Disease where it promotes uptake and colonization of small intestine epithelium by adherent, invasive pathogenic *E. coli* (Barnich et al. 2007).

Expression of CEACAM6 and closely related CEACAM5 is deregulated and overexpressed in cancers of colorectal epithelium, with surface levels inversely correlated with both the degree of colonocyte differentiation (Kuroki et al. 1999) and positive clinical outcome (Jantscheff et al. 2003). The two CEACAMs are also expressed in a high proportion of tumor cell lines derived from breast, ovary, pancreas, prostate, and lung (Blumenthal et al. 2007; Beauchemin and Arabzadeh 2013). It has been proposed that CEACAM5/6 overproduction has a causative role in tumorgenesis, acting via an imbalance of cell surface adhesion molecules that disrupts differentiation, inhibits apoptosis and promotes both tumor formation and metastases (Ilantzis et al. 1997; Ordonez et al. 2000)

Earlier studies identified CEACAM6 immunoreactivity in normal adult lung, with localization to both alveolar and airway epithelium (Tsutsumi et al. 1990; Scholzel et al. 2000). Recently, we confirmed that CEACAM6 is expressed by a subpopulation of alveolar and airway epithelial cells of infant and adult human lung, and we found that the fully glycosylated protein is secreted into lung lining fluid where it binds to surfactant and protects from inhibition by extraneous proteins in vitro (Kolla et al. 2009; Chapin et al. 2012). Production appeared to be up‐regulated during neonatal lung disease, perhaps related to roles of CEACAM6 in surfactant function, cell proliferation and innate immune defense.

The CEACAM6 gene is not present in rodents, and its emergence in primates may represent pathogen‐host co‐evolution, providing a protein capable of binding bacteria specific for primates. In order to explore the role of CEACAM6 in vivo, Chan and Stanners (Chan and Stanners 2004) developed a transgenic mouse (CEABAC) using a human BAC containing the genes for human CEACAMs 3, 5, 6, and 7. Similar to the expression profile in humans, the CEABAC mouse expressed immunoreactive CEACAM6 in a number of tissues including lung. In this study we have further characterized expression of human CEACAM6 in lung of CEABAC animals and examined effects of different types of lung injury. We hypothesized that CEACAM6 expression increases during the repair phase after lung injury and is a marker of proliferating progenitor cells that replenish the alveolar epithelium. Our results demonstrate up‐regulated expression of CEACAM6 after bleomycin, LPS and hyperoxic lung injury and support the proposal that CEACAM‐6 expressing cells can differentiate into alveolar type I and type II cells.

## Materials and Methods

### Animals

CEABAC transgenic mouse line 1747 (FVB background) was obtained from Clifford P. Stanners (McGill University, Montreal, Quebec, Canada). The mouse was constructed using human bacterial chromosome (Genbank Accession No. BC627193, Research Genetics Inc, Huntsville, AL) containing part of the human CEA family gene cluster which includes the complete CEACAM5, CEACAM3, CEACAM6, and CEACAM7 genes. We confirmed expression of these genes in the lung by RT‐PCR utilizing published primer sequences (Chan and Stanners 2004). In our studies, we used heterozygous mice obtained by breeding to FVB animals; wild‐type (wt) littermates were used as controls. For identification and sorting of type II cells, we crossed CEABAC mice with a transgenic mouse line referred to as the CBG mouse, which is short for SP**C**‐**B**AC‐**EGFP**. The CBG mouse line was developed, using a BAC vector RT23‐247J9, modified by insertion of an IRES‐EGFP cassette into the 3′UTR of the SP‐C gene, which is centrally positioned on a 181 K bp segment of genomic DNA. By fluorescence microscopy, lungs of CBG mice express enhanced green fluorescent protein (EGFP) in virtually all type II cells (Vanderbilt et al. 2015). The Committee on Animal Research of the University of California, San Francisco, approved all studies, and all procedures conformed to the NIH Guide for the Care and Use of Laboratory Animals. Mice were housed in the Laboratory Animal Resource Center (LARC) barrier facility, which is maintained at ambient temperature and humidity.

### Human samples

Samples of human infant postmortem lung tissue were obtained from the Department of Pathology, Children's Hospital of Philadelphia under IRB‐approved protocols. Tracheal aspirate samples from intubated premature infants were collected as part of a previous, IRB‐approved clinical trial and stored at −80°C.

### Mouse genotyping

DNA was extracted from mouse tails using 20 *μ*g Proteinase K (Ambion, Grand Island, NY) diluted in 50 mmol/L Tris HCl, 20 mmol/L NaCl, 1 mmol/L EDTA, and 1% SDS, pH 8.0. DNA was amplified using Phusion Hot Start DNA polymerase (Thermo Scientific, Pittsburgh, PA) using an Eppendorf Mastercycler Nexus Gradient (Eppendorf, Hamburg, Germany). Primers for CEACAM5 and EGFP were purchased from Eurofins Scientific (Petaluma, CA). The forward primer for CEACAM5 was 5′‐ GAAATGACACAGCAAGCTAC ‐3′, the reverse primer was 5′‐ ATAGACTGTGATCGTCGTGA‐3′. The forward primer for EGFP was 5′‐TGAAGTTCATCTGCACCACCG‐3, the reverse primer was 5′‐TGATGCCGTTCTTCTGCTTGTC‐3′.

### Surgical procedures for instillation

Animals were given preemptive Buprenorphine (0.1 mg/kg, IP) and anesthetized with continuous 3% Isoflurane throughout the surgery. All survival procedures were done in a Biosafety Cabinet, using the aseptic technique. The trachea was exposed and either saline, Bleomycin (*Streptomyces verticillus* Bleomycin Sulfate 0.05 units per mouse, Sigma B5507, St. Louis, MO) or LPS (*Escherichia coli* Lipopolysaccharide 50–500 *μ*g per mouse; Sigma L2880) was instilled into the trachea between the cartilaginous rings using a 24G insulin syringe. For bleomycin instillation, we included 10% colloidal carbon, which allowed visual postmortem identification of areas of instillate deposition (and bleomycin injury). The mice were allowed to fully recover from the anesthesia prior to being placed back in the animal room. Mice were weighed daily and those that lost more than 25% of their original body weight were sacrificed. At 1–28 days, postinstillation animals were euthanized with 0.5 mL Euthasol^®^ (390 mg/mL Sodium Pentobarbital, Virbac Animal Health, Fort Worth, TX) diluted 1:20 with sterile saline followed by production of bilateral pneumothoraces.

### Hyperoxia

CEABAC and wt littermates (pn day 3) were either exposed to normoxia or hyperoxia for 7 days at 80% O_2_. On the 7th day, mice exposed to hyperoxia were gradually weaned to normoxia over a 4‐h period. During the experiment, dams in hyperoxia were traded daily with dams at normoxia and vice versa. At 3–5 days postexposure animals were euthanized and the lungs were inflation fixed and processed as described below. There were 5 CEABAC and 4 wt mice in the hyperoxia group and 3 CEABAC and 2 wt mice in the normoxia group.

### Tissue and bronchial alveolar lavage collection

Lungs were harvested from the mice by opening the chest and placing a 24G angiocatheter (needle removed) into the trachea and securing with a suture. The left lung was tied off, removed, weighed and snap frozen in liquid nitrogen and stored at −80°C until used for protein or RNA isolation. The right lung was fixed with 4% paraformaldehyde for 1 h at 20 cm of water pressure in situ, placed in Phosphate Buffered Saline (PBS) for 15–30 min, and transferred to 30% sucrose in PBS overnight. Individual lobes were frozen in O.C.T Compound (Tissue‐Tek 4583, Torrance, CA) and stored at −80°C until used for cryosectioning. Bronchial alveolar lavage (BAL) was performed on some mice using a 1 cc of PBS reinstilled into the lung 3 times. The BAL was then immediately processed.

### Processing of BAL samples

After centrifugation at 500 × g for 5 min to remove cells, BAL supernatant was stored at ‐80°C in the presence of protease inhibitors and subsequently centrifuged at 27,000 × g for 60 min to isolate large aggregate surfactant pellet and supernatant fractions. Large aggregate pellet was resuspended in surfactant buffer (10 mmol/L Tris, 154 mmol/L NaCl, 1.5 mmol/L CaCl_2_, pH 7.4) and an aliquot was extracted (Bligh and Dyer 1959) and assayed for phospholipid (PL) content (Bartlett 1959). Total protein was measured on both fractions using QuantiPro BCA assay system (Sigma, St. Louis, MO).

### CEACAM6 content

Analysis of human CEACAM6 was performed using previously described methods with slight modifications for the presence of mouse immunoglobulins (Chapin et al. 2012). Western analyses used mouse anti‐human CEACAM6 primary antibody (Carcinoembryonic Cell Adhesion Molecule 6;Clone 9A6 1:1000; Santa Cruz Biotech, Santa Cruz, CA), which is specific for CEACAM6, and anti‐mouse HRP‐labeled TrueBlot^®^ secondary antibody (1:1000, Rockland Immunochemicals Inc., Gilbertsville, PA), which avoided detection of mouse IgG heavy and light chains. The blots were developed using ECL Plus (GE Healthcare Life Sciences), visualized by autoradiography and quantified using the Storm 840 scanner equipped with a blue fluorescence/chemi‐fluorescence detector and Imagequant software (GE Healthcare Life Sciences). All blots were run with recombinant human CEACAM6 (R & D Systems Inc., Minneapolis, MN) and adult human lung homogenate as internal controls. Equivalent protein loading and transfer between lanes was determined by staining blots with 0.1% amido black.

CEACAM6 in BAL was quantified with an immunodot assay using serial dilutions of denatured recombinant CEACAM6 (rCEACAM6, R & D Systems) as the standard. BAL large aggregate surfactant pellet samples containing 1.33 *μ*g of total pellet protein were added to PBS and serially diluted as for the standard and aliquots were applied to 0.2 *μ*m nitrocellulose membrane in a 96‐well, dot‐blot manifold. All blots were run with two internal control samples of known CEACAM6 concentration to evaluate reproducibility of the assay. After blocking with non‐fat milk solution, the blot was incubated with mouse anti‐human CEACAM6 (1:1000 dilution) and subsequently with secondary antibody conjugated to horseradish peroxidase (HRP). Values for each sample that fell on the linear portion of the standard curve were averaged and expressed as percent of protein or percent of PL. The assay has a fivefold linear range with an intra‐ and interassay variability of 5% and 7%, respectively.

### Immunofluorescence staining

3 μm cryosections of fixed lung were cut using a Leica CM1850 cryostat (Leica Microsystems Inc., Buffalo Grove, IL). Primary antibodies used in this study were anti‐human CEACAM6 (1:100; Clone 9A6, Santa Cruz Biotech, Santa Cruz, CA), anti‐mouse OTS‐8 (Type I alveolar epithelial cell marker; 1:200; Clone 8.1.1, Developmental Studies Hybridoma Bank, University of Iowa, IA), anti‐mouse AQP5 (Aquaporin 5, Type I alveolar cell marker; 1:200, Abcam ab78486, Cambridge, MA), anti‐sheep SP‐B (Type II/Clara cell marker a kind gift of Sam Hawgood (Joe et al. 1997), anti‐mouse *α*‐SMA (alpha‐smooth muscle actin; 1:400, Abcam ab5694), anti‐mouse Vimentin (mesenchymal cell marker; 1:50, Santa Cruz Biotech sc‐7557‐r), anti‐mouse CD45 (pan‐nucleated hematopoietic cell marker; 1:100, BD553076, BD Biosciences, San Jose, CA), and anti‐mouse CGRP (calcitonin gene‐related peptide, pulmonary neuroendocrine cell marker; 1:500 C8198, Sigma, St. Louis, MO). Secondary antibodies, tagged with Alexa 488 or Alexa 594 were used at 1:1000 (Invitrogen, Grand Island, NY). Sections were permeablized with PBS containing 0.3% Triton‐X 100 and 1% bovine serum albumin (PBS+), blocked with 10% normal goat serum in PBS+, incubated in Mouse on Mouse IgG blocking reagent (M.O.M.; Vector labs, Burlingame, CA) followed by incubation in primary antibody diluted in PBS+ containing 0.5% nonfat dry milk for 60 min at room temperature or 4°C overnight. After extensive washing, sections were incubated in secondary antibody for 30 min at room temperature, mounted using Prolong Gold anti‐fade reagents with DAPI (Molecular Probes, Eugene, OR) and images were obtained using a Leitz Orthoplan 2 microscope (Leica Microsystems, Bannockburn, IL). Each fluorescent image was captured in a separate channel using a Leica DC500 digital camera. For bleomycin‐exposed lungs, the intensity and frequency of CEACAM6 staining were evaluated at various time points after instillation by a blinded observer using a semi‐quantitative scale. In some instances images were obtained using a Nikon Ti‐E inverted spinning disk microscope (Nikon Instruments Inc., Melville, NY) and Micro‐Manager software (an open source software program) at the UCSF Gladstone Institute's Nikon Imaging Center with the assistance of Kurt Thorn, Ph.D., Director of the Nikon Imaging Center at UCSF.

### Semiquantitative analysis of cell immunostaining

Lung cryosections from mice exposed to hyperoxia and normoxia were stained for CEACAM6, AQP5 and DAPI as described above. Four blinded, independent observers counted CEACAM6 positive cells from 6 sections (10 randomly chosen fields, 20× magnification) using either Photoshop (Adobe systems, San Jose, CA) or Image J (National Institutes of Health). Using modified methods described by Ochs (Ochs 2014), point counts of alveolar epithelium that intersected horizontal or vertical lines were made by using a grid with 13 horizontal and 11 vertical lines overlaying phase images from the same fields used for counting CEACAM6^+^ cells. Edges were not counted to avoid edge effect (Knudsen et al. 2010).

### Plastic thin sections

Lungs were fixed by intratracheal instillation of 2% glutaraldehyde/1% paraformaldehyde in 0.1 mol/L sodium phosphate buffer (pH 7.4; 2 h at room temperature) and then postfixed overnight in 1.5% osmium tetroxide suspended in Veronal acetate buffer at 4°C. They were stained *en bloc* in 1.5% uranyl acetate in maleate buffer and then quickly dehydrated in ice‐cold acetone and propylene oxide. The tissue was finally infiltrated and embedded in LX 112 (Ladd Research Industries, Burlington, VT). 0.5 *μ*m sections were cut with a microtome (Leica Ultra Cut ULT, Leica Microsystems Inc., Buffalo Grove, IL) and stained with toluidine blue. Images were obtained as with cryosections.

### Fluorescence activated cell sorting (FACS)

CEABAC lung cells from untreated or bleomycin‐treated mice were isolated by elastase digestion and sorted as previously described for rat alveolar epithelial cells (Gonzalez et al. 2009). Briefly, cells were initially treated with human IgG (50 *μ*g/mL; Sigma) to block non‐specific binding of mouse monoclonal primary antibody. CEACAM6 (clone 9A6) antibody was directly labeled with Alexa 488 using Zenon technology (Invitrogen, as recommended). Cells were stained with labeled CEACAM6 antibody (60 *μ*L) and rat anti mouse CD11b (1:50) to label cells of myeloid origin (AbD Serotec, MCA74G, Raleigh, NC). Cells were sorted and collected on an Aria II FACS instrument (BD Biosciences, San Jose, CA). In some cases collected cells were sorted directly into RLT Bufffer (Qiagen Inc, Valencia, CA) for future use, while in other cases cells were fixed in 50% ethanol for cell cycle analysis and stained with a solution containing propidium iodide (PI, 20 mg/mL), DNase‐free RNase A (0.2 mg/mL), and 0.1% Triton‐X100 in calcium, magnesium‐free PBS. All analyses were done using Flow Jo software (Tree Star Inc., Ashland, OR).

### q‐PCR

Total RNA was prepared from lung tissue using Qiagen RNeasy isolation kit with DNase digestion (Valencia, CA). RNA was reverse transcribed using Ambion RETROscript kits (Applied Biosystems/Ambion, Austin, TX). Quantitative real‐time PCR (q‐PCR) amplification of cDNA was performed using an ABI PRISM 7700HT Sequence Detector System with a 384‐well block (Applied Biosystems‐Invitrogen, Foster City, CA). Reaction mixtures consisted of 2 ng cDNA, TaqMan 2X Universal PCR Master Mix (Applied Biosystems‐Invitrogen), forward primer, reverse primer and probe (see below) in a reaction volume of 10 *μ*L. Using a two‐step PCR program, we heated samples to 95°C for 10 min followed by 40 cycles at 95°C for 15 sec and 60°C for 1 min. Differences in the amount of cDNA amplified were corrected by normalization to endogenous mouse genes Polr2a and Ipo8. Standard curves for test genes and housekeeping genes were constructed on each plate from serial log dilutions of stock whole lung cDNA; the relative quantification in triplicate for each experimental sample was obtained by the standard curve method. Control reactions were performed in the absence of target DNA. Primer/probes sets were purchased from Applied Biosystems‐Invitrogen. The following primer/probe sets were used: human CEACAM6 Hs03645554_m1, human CEACAM5 Hs00944025_m1, mouse Sftpc Mm00488144_m1 and mouse Aqp5 Mm00437579_m1, mouse Ipo8 Mm01255158_m1, and mouse Polr2a Mm00839493_m1.

### Statistical methods

Numerical data are expressed as mean ± SD and analyzed by Student's *t* test for normally distributed data.

## Results

### Pulmonary expression of human CEACAM6 in CEABAC mice

We chose the CEABAC transgenic model for our studies based on the original observations of Chan and Stanners (Chan and Stanners 2004) that human CEACAM6 was expressed in lung tissue by Western analysis, albeit at a low level compared to bone marrow and epithelium of vaginal and gastrointestinal tissues. By immunohistochemistry, they observed CEACAM6 staining in trachea and bronchial epithelium plus scattered alveolar cells. The 187 kb human BAC used in generation of this mouse contains genes for CEACAM3, CEACAM5, CEACAM6 and CEACAM7; line CEABAC‐1747, which we used, contains 10 copies of the transgenes (Chan and Stanners 2004). All experiments were performed with CEABAC heterozygous animals obtained by breeding to wt FVB mice.

**Figure 1:**
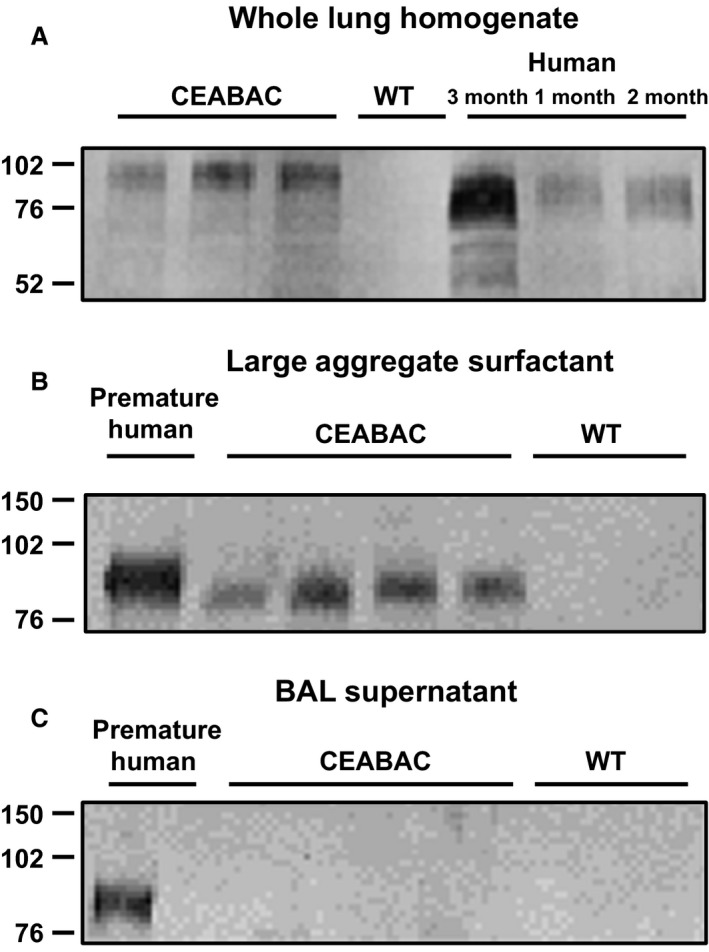
Representative Western blot analyses of lung CEACAM6 in adult CEABAC mice compared to human infants. (A) Whole lung homogenate. 10 *μ*g protein was loaded for each sample. Bands are observed at approximately 90, 70, and 50 kDa for most CEABAC and term human infant samples but not in wt mice. CEACAM6 in CEABAC mice consistently ran at slightly higher molecular weight than human lung CEACAM6, probably reflecting different amount of glycosylation. By scanning densitometry, total CEACAM6 signal is similar for CEABAC mice and human lungs. (B) Large aggregate surfactant fraction of BAL. 2 *μ*g protein was loaded for mouse samples and 0.1 *μ*g for a sample from an intubated premature human infant. A single 90 kDa band is observed for CEABAC samples and human surfactant but not wt mouse samples. (C) Supernatant of samples from B. 5 *μ*g protein was loaded for mouse samples and 1 *μ*g for the human. CEACAM6 was detected in the human specimen as previously reported (Chapin et al. 2012) but not in mouse samples.

Initial experiments further characterized expression of CEACAMs in lungs of CEABAC mice. By RT‐PCR, we detected transcripts for all 4 human CEACAMs in lung tissue, with much weaker signal for CEACAM7 (data not shown); previously, Chan and Stanners (Chan and Stanners 2004) reported that CEACAM3 and CEACAM7 were not detected in any tissues by immunoblotting. Further studies focused on expression of CEACAM6 in lung parenchyma as a model for our previous observations that this protein has multiple functions in human lung alveoli (Kolla et al. 2009). By Western analysis (Fig. 1A), lung tissue of adult CEABAC mice contains immunoreactive CEACAM6 with a major band at ~90 kDa and minor bands at ~70 and 50 kDa, whereas no signal is detected in wt animals. The level of CEACAM6 expression is similar to that observed in lung specimens from human infants (without lung disease) and about one‐third of the level in adult human lung (Kolla et al. 2009), supporting the relevance of CEABAC mice as a model. CEACAM6 was present in large aggregate surfactant isolated from BAL of adult CEABAC mice (Fig. 1B) as previously observed for tracheal aspirate of intubated premature human infants. The level of surfactant‐associated CEACAM6, expressed as percent of total protein in the fraction, was ~10‐fold less in CEABAC mice than in surfactant from an infant with lung disease (lane 1, Fig. 1B). CEACAM6 was not detected in the supernatant fraction of BAL of CEABAC mice after removal of large aggregate surfactant, whereas the protein was present in human supernatant as previously reported (Chapin et al. 2012). We speculate that this species difference for relative level of CEACAM6 in surfactant and supernatant fractions reflects in part the higher amount of alveolar/airway CEACAM6 in infants with lung disease, compared to healthy CEABAC mice, and limited CEACAM6 binding capacity of surfactant lipids.

**Figure 2:**
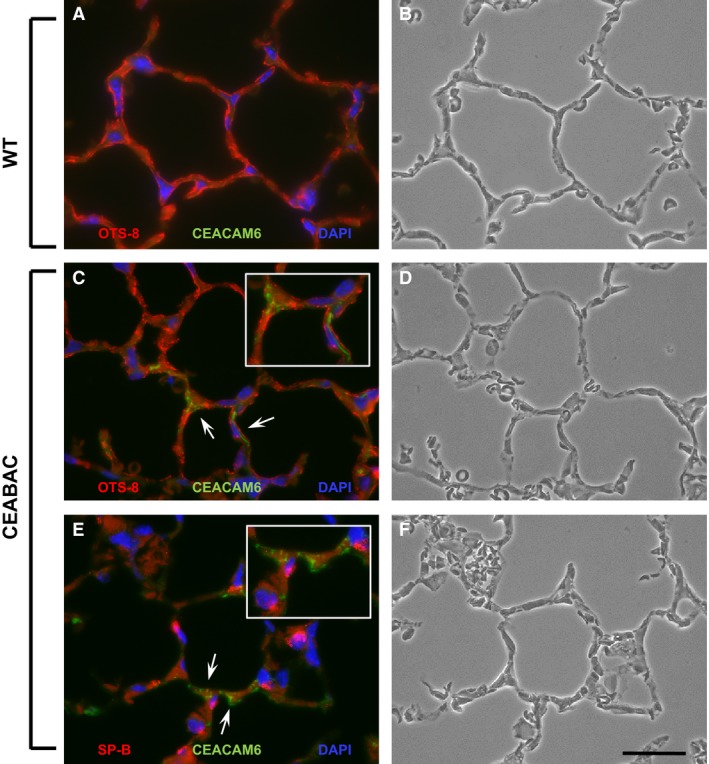
Immunohistochemistry of CEACAM6 in cryosections of lung from adult wt and CEABAC mice. (A) Immunostaining of wt mouse lung. OTS‐8 red staining shows alveolar type I cells, blue nuclei (DAPI) and absence of CEACAM6 (green). (b) Corresponding phase contrast image. (C) OTS‐8 and CEACAM6 immunostaining of CEABAC lung. Relatively low intensity CEACAM6 staining (green) is observed in some alveoli and does not appear to co‐localize with OTS‐8 as a marker of type I cells. (D) Corresponding phase contrast image. (E) SP‐B and CEACAM6 immunostaining (red) of CEABAC lung. Low intensity CEACAM6 staining (green) is observed in some alveoli and does not appear to co‐localize with SP‐B (red) as a marker of type II cells. (F) Corresponding phase contrast image. Insets in c and d show ~ twofold magnified views of CEACAM6^+^ alveolar regions (arrows). Images are representative of multiple sections from lungs examined. Bar = 20 *μ*m.

Immunohistochemistry for parenchymal CEACAM6 was examined in cryosections of inflation‐fixed lungs of mice using CEACAM6 antibody, OTS‐8 (mouse podoplanin) to identify type I cells, and anti‐SP‐B to mark type II cells. No CEACAM6 signal was detected in wt animals as expected (Fig. 2A,B). In CEABAC mice, CEACAM6 immunoreactivity was detected in ~10% of alveoli, which could reflect very low expression and the sensitivity of the immunostaining approach. In a qualitative survey of sections, co‐localization of CEACAM6 with either OTS‐8 (Fig. 2C,D) or anti‐SP‐B (Fig. 2E,F) in the alveolar epithelium was observed very rarely (not shown). Positive staining appeared to be epithelial (see higher power insets), consistent with findings in other CEABAC mouse tissues (Chan and Stanners 2004) and human lung (Chapin et al. 2012), and with the presence of secreted CEACAM6 in BAL of CEABAC animals (see Fig. 1B). Rarely, a CEACAM6^+^ cell was observed in the interstitium (not shown), which may represent lymphoid cells.

### Bleomycin‐induced lung injury increases CEACAM6 expression

**Figure 3:**
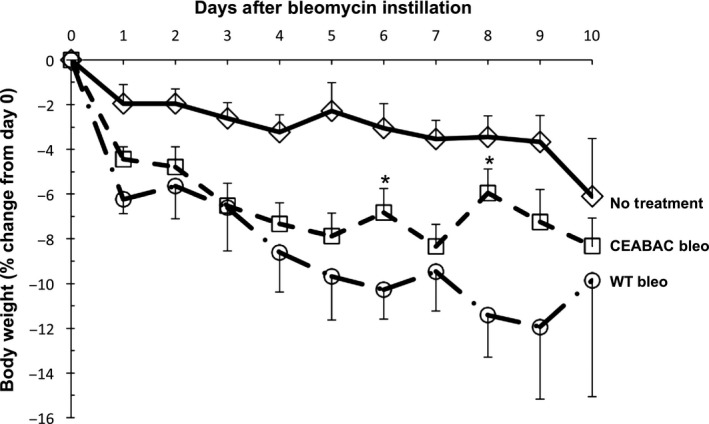
Change in body weight over time for mice with and without instillation of bleomycin. Data are % change from baseline (day 0) as mean ± SE for 6 experiments with 15 untreated littermate CEABAC and wt mice (diamond), 33 CEABAC mice receiving bleomycin (square), and 13 wt mice given bleomycin (circle). There is progressive weight loss for wt animals given bleomycin. Weight loss for bleomycin‐treated CEABAC mice appears to stabilize after 3 days and is significantly different from wt bleomycin mice on days 6 and 8 (*, *P* < 0.05).

We initially used tracheal instillation of bleomycin to test the effect of lung injury on CEACAM6 expression, choosing a low lethality dose (0.5 units/animal) to allow for recovery and repair. All studies (*n* = 120 mice) were performed at about 3 months of age with assessment between 3 and 21 days postinstillation. As shown in Figure 3, bleomycin instillation to wt mice decreased weight by a maximum of 12% over 10 day compared to <6% in control animals without surgery or instillation; surviving bleomycin‐treated animals increased their weight to baseline over the next 2 weeks (data not shown). CEABAC mice lost weight initially after bleomycin similar to wt animals, presumably reflecting both surgical stress and acute bleomycin injury, however the weight of CEABAC mice tended to stabilize between 3 and 10 days and was significantly different from bleomycin‐treated wt mice at 6 and 8 days. Overall mortality was low (4 of 62 animals receiving bleomycin), reflecting the dose chosen to allow injury with survival. Left lung weight/body weight (mean/SD) was 17.1 ± 1.3 mg/g for uninjured animals; 10 days after bleomycin injury the ratio was increased nearly twofold and not different between wt (32.5 ± 8.8, *n* = 6) and CEABAC (31.8 ± 6.0 mg/g, *n* = 11) mice. Ten days after bleomycin injury, total protein in BAL was significantly increased four to fivefold compared to uninjured animals and was not different between wt (0.915 mg, *n* = 4) and CEABAC (1.215 mg, *n* = 11) mice. Overall, these data do not support a protective role for human CEACAM6 related to initial severity of lung injury after bleomycin exposure in this model.

**Figure 4:**
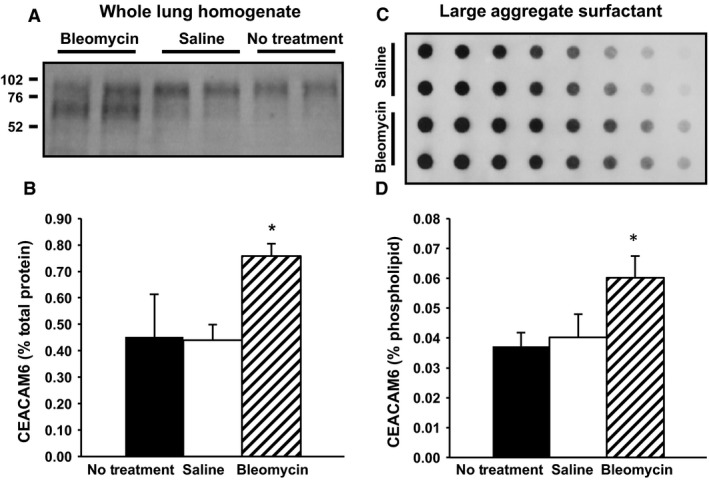
Effect of bleomycin and saline on CEACAM6 content in lung tissue and lavage surfactant at 10 days. (A) Representative Western blots of lung homogenate. Equal amounts of total protein were loaded. Compared to No Treatment and Saline, increased total CEACAM6 signal is observed for lungs of bleomycin‐treated animals. (B) CEACAM6 content of homogenate by scanning densitometry (mean ± SD). (C) Representative immunodot blot for large aggregate surfactant (with duplicate rows containing serial dilutions of equal amounts of PL/well). Increased signal is observed for bleomycin versus saline that is most evident at higher dilutions. (D) CEACAM6 content of surfactant by scanning densitometry. Data are mean ± SE, and *n* = 5; *P < 0.05 versus saline treatment.

In Western analysis of lung homogenate, CEACAM6 was increased 1.7‐fold in bleomycin‐treated vs saline‐instilled mice at 10 days (Fig. 4, left panels); the increase is most prominent for the 70 kDa band, which may reflect partially glycosylated protein. There was a similar 1.6‐fold increase in amount of CEACAM6 associated with surfactant after bleomycin injury as assessed by immunodot blot assay (Fig. 4, right panels). The relatively modest increase in CEACAM6 in whole lung homogenate compared to the marked increase in immunostaining intensity likely reflects the patchy nature of bleomycin injury with sparing of many parenchymal areas. The bleomycin‐induced increase in CEACAM6 was also reflected at the transcript level (Table 1), consistent with activation of gene expression. CEACAM6 mRNA content increased 2.1‐fold with a 2.7‐fold elevation for CEACAM5, a closely related protein that is encoded by a gene adjacent to CEACAM6 on chromosome 19. We could not assess effects of bleomycin on CEACAM5 protein content because of the unavailability of an anti‐CEACAM5 antibody that does not cross react with CEACAM6. By contrast, there was no effect of bleomycin on levels of AQP5 and SFTPC mRNAs as markers for type I and type II cells, respectively (Table 1).

**Table 1:** mRNA content in lungs of bleomycin‐treated and untreated CEABAC mice

Gene	*n*	Bleomycin	Control	Fold change	*P*
CEACAM6	7/3	0.47 ± 0.10	0.22 ± 0.13	2.1	0.005
CEACAM5	7/3	0.98 ± 0.57	0.36 ± 0.19	2.7	0.04
AQP5	3/3	1.14 ± 0.54	0.99 ± 0.05	1.1	NS
SFTPC	3/3	122.7 ± 27.7	124.2 ± 4.0	1.0	NS

QPCR analysis of mRNA content of in lungs of bleomycin‐treated mice 10 days postinstillation compared to control animals. Values are means ± SD; data are normalized to Ipo8 and Polra2.

**Figure 5:**
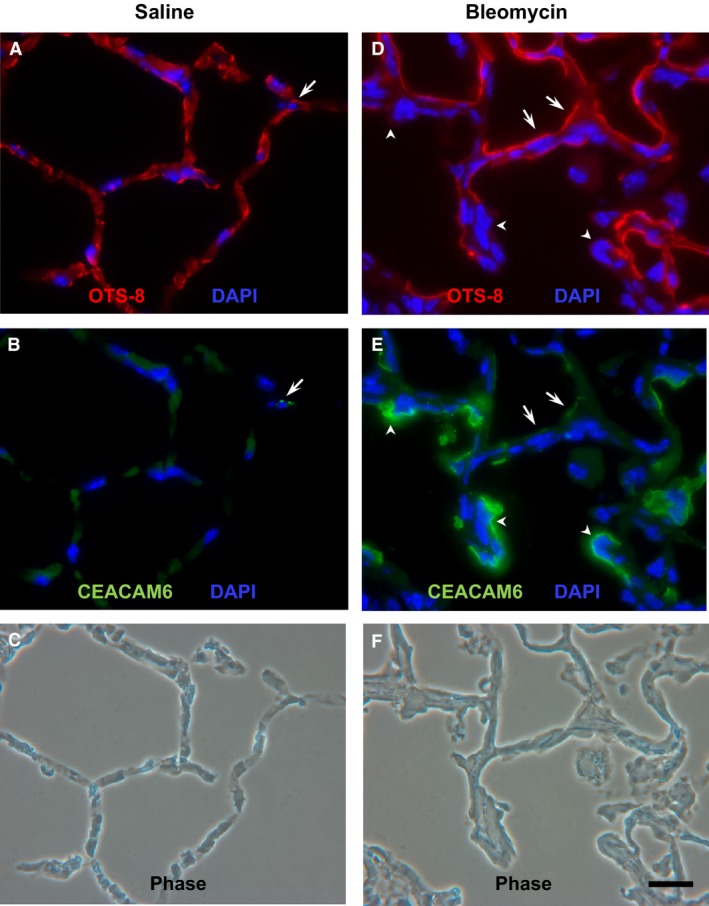
Immunohistochemistry for CEACAM6 expression in cryosections of lung from adult CEABAC mice treated with saline (A–C) or bleomycin (D–F) for 10 days. (A) OTS‐8 staining in saline‐instilled lung. (B) punctate CEACAM6 staining (green, arrow) of one cell that is immunopositive for OTS‐8. (C) phase contrast image corresponding to A,B. (D) OTS‐8 staining in bleomycin‐instilled lung. (E) CEACAM6 staining of multiple cells that are either positive (e.g., arrows) or negative (e.g., arrowheads) for OTS‐8. (F) phase contrast image corresponding to d and e; note thickened interstitium compared to saline (C), reflecting bleomycin injury. Bar = 20 *μ*m. All fluorescence images were obtained at the same exposure settings.

**Figure 6:**
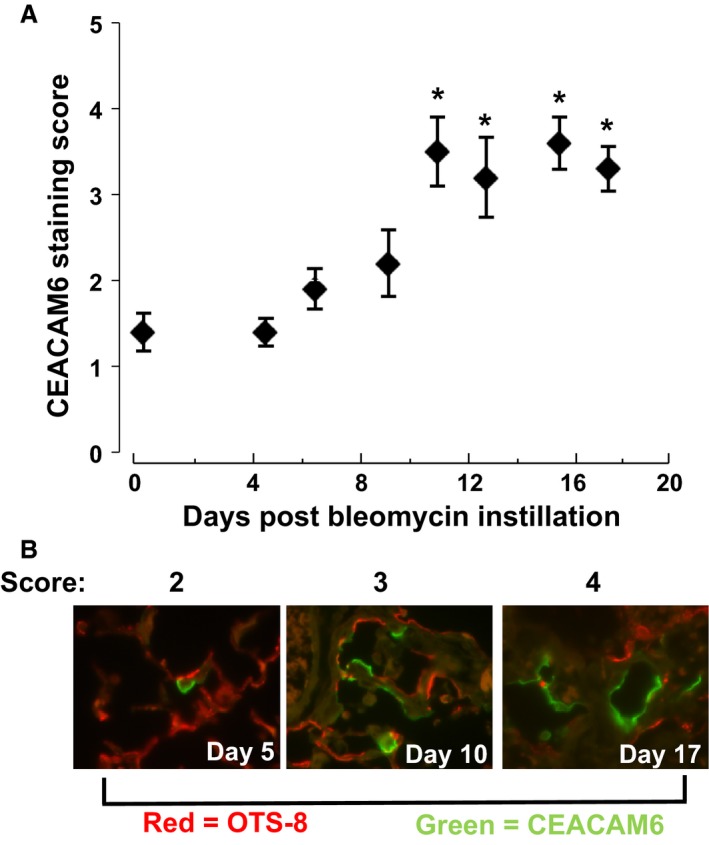
CEACAM6 staining score for lungs after bleomycin instillation. A, score for cryosections of uninjured lungs (day 0) and at days 5–19 after bleomycin instillation (mean ± SE for 10 fields on each of 2 sections for lungs of 8 mice). (B) representative images for scores of 2, 3 and 4. The score is a composite of staining intensity and number of positive cells per field by a blinded observer. Maximal signal occurs by day 11; *P < 0.05 versus day 0. Results from a second experiment were similar.

Figure 5 shows immunostaining for CEACAM6 and OTS‐8 10 days after instillation of saline (left panels) or bleomycin (right panels) to CEABAC mice. Saline‐treated animals retain normal alveolar architecture by phase contrast (panel C) and demonstrate low levels of CEACAM6 immunoreactivity as found in untreated CEABAC mice (see Fig. 2); there is apparent co‐localization of CEACAM6 and OTS‐8 in one cell. By contrast, bleomycin instillation markedly increased CEACAM6 signal (panel E) in injured areas of lung (as shown by phase contrast in panel F); uninjured areas did not have increased CEACAM6 signal (not shown). Relatively faint CEACAM6 immunostaining appears to co‐localize with OTS‐8 in some cells (arrows), whereas all other cells in the field show intense CEACAM6 signal without OTS‐8 staining (e.g., arrow heads). These results indicate that lung injury with bleomycin increases both the number of cells expressing detectable CEACAM6 and the level of expression per cell. We used an immunohistochemical scoring system as described in Methods to assess the time course of increased CEACAM6 expression after bleomycin instillation. As shown in Figure 6A, increased CEACAM6 immunostaining is first detected on day 7 and plateaus between 11 and 19 days; part B of Figure 6 shows representative images and scoring. Similar results were found in a second experiment. Thus, increased CEACAM6 expression occurs in a subset of alveolar epithelial cells during the repair phase after bleomycin lung injury.

**Figure 7:**
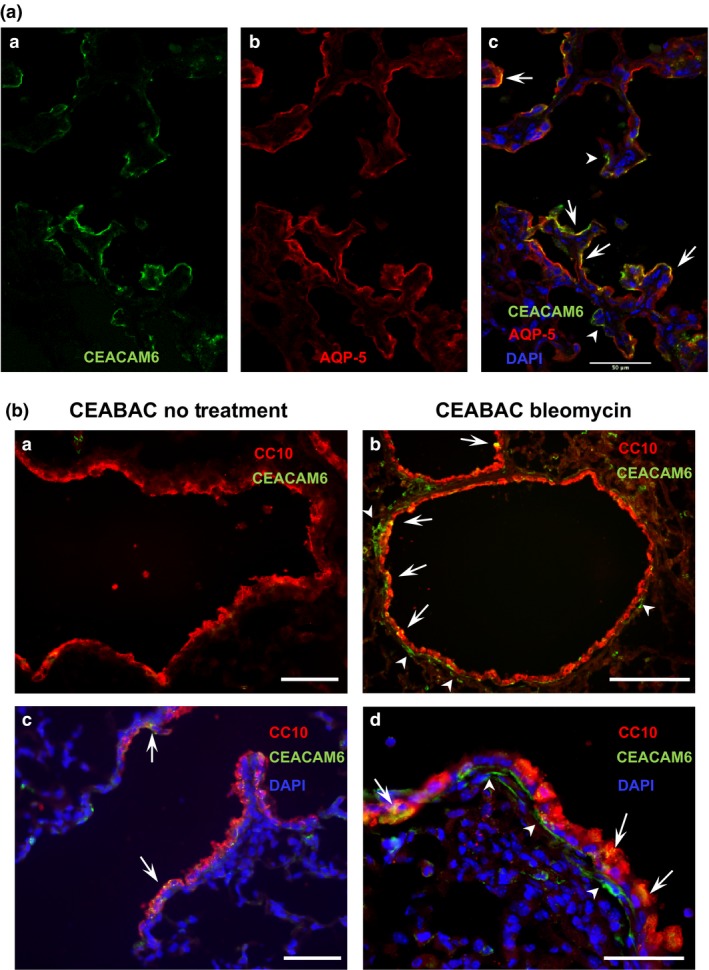
Increased CEACAM6 immunostaining in epithelial cells after bleomycin instillation. Panel A, A–C, co‐localization of CEACAM6 and AQP5 in alveolar epithelium. By confocal imaging, some alveolar cells co‐stain (yellow) for CEACAM6 (green) and AQP5 (red, e.g., arrows) and other CEACAM6^+^ cells are negative for AQP5 (e.g., arrow heads). Bar = 50 *μ*m. Panel B, CEACAM6 and CC10 staining in airways of control (A,C) and bleomycin‐treated (B,D) CEABAC mice. Most cells of control animals are CC10‐positive with sparse CEACAM6 signal (A and C—higher power). After bleomycin (B,D) co‐localization of CEACAM6 and CC10 is observed in airway epithelial cells (arrows) and CEACAM6 signal occurs basal to the epithelium. Bars = (A) 40 *μ*m; (B) 100 *μ*m; (C) and (D) 40 *μ*m.

To confirm the finding of apparent increased co‐localization of CEACAM6 and OTS‐8 after bleomycin injury (see Fig. 5D,E), we performed immunostaining and confocal imaging with an antibody to AQP5 (another type I cell marker). As shown in Figure 7A, some cells (arrows) co‐express the two antigens while other cells (arrow heads) are CEACAM6^+^ but AQP5‐negative, similar to the findings with OTS‐8. In studies performed at 28 days after bleomycin injury, the number and staining intensity of CEACAM6^+^ cells was markedly reduced compared to day 10 and co‐localization with OTS‐8/AQP5 was rarely observed (data not shown). We also examined lung airways for CEACAM6 staining with and without bleomycin using CC10 as an epithelial marker. There was occasional apparent co‐localization of CEACAM6 and CC10 in airway epithelium of control lung (arrows in Fig. 7B, panel C). In bleomycin‐treated lungs, co‐localization in the airway epithelium was increased (arrows, panels B and D), and there was appearance of generalized CEACAM6 staining basal to the CC10^+^ cells (arrowheads). Thus, bleomycin injury increases CEACAM6 expression in both alveolar and in airway epithelium and basal cells. By contrast, no co‐localization was observed in lung parenchyma for CEACAM6 and *α*‐SMA (smooth muscle cells), vimentin (fibroblasts), CD45 (lymphoid cells) and CGRP (neuroendocrine cells) (Figure S1).

**Figure 8:**
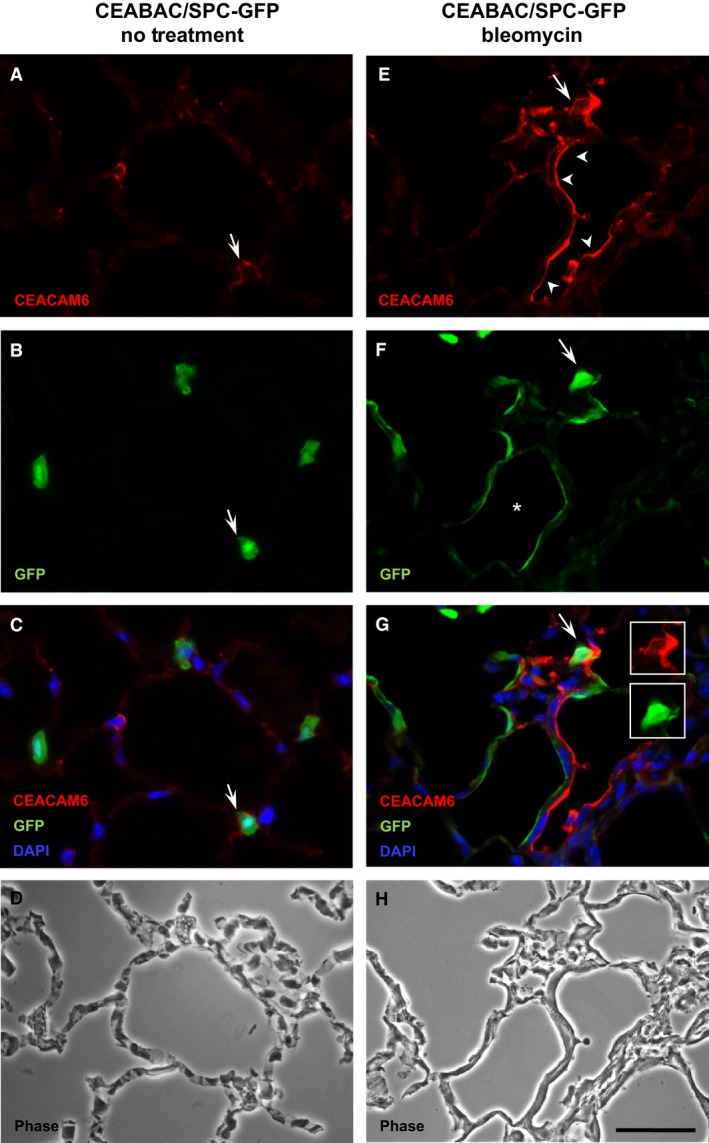
CEACAM6 and EGFP fluorescence signal in control and bleomycin‐treated CEABAC/CBG mice. In control animals (A–D), cuboidal type II cells (green) are evident along with 2 CEACAM6^+^ (red) cells, with one example of co‐localization (arrow). In bleomycin‐treated mice (E–H), there is increased CEACAM6 signal (red) in injured areas and altered cell shape: F versus B, shape of many EGFP ^+^ cells is flattened after bleomycin (arrowheads) with one cell showing apical CEACAM6 and cytoplasmic EGFP signal (G—arrow and inset); Phase micrographs are shown in D and H. Bar = 40 *μ*m. * alveolus with EGFP ^+^ epithelium.

To specifically examine CEACAM6 expression in type II cells, which can differentiate into type I cells during development and repair from injury (Kapanci et al. 1969; Barkauskas et al. 2013), we crossed CEABAC mice with CBG transgenic mice: the CBG mouse (SP**C**‐**B**AC‐**EGFP**) contains a mouse BAC modified to express EGFP within the mouse SP‐C gene 3′UTR. In the CBG mouse ~99% of type II cells express EGFP (Vanderbilt et al. 2015). In uninjured CEABAC/CBG mice, cells positive for EGFP are all cuboidal and apparent co‐localization of EGFP (green) and CEACAM6 (red) staining was occasionally observed as shown in Figure 8 (arrow in panel C); most EGFP^+^ type II cells were negative for CEACAM6 under the conditions used. Ten days after bleomycin, some EGFP^+^ cells of injured areas were cuboidal (e.g., Fig. 8, arrow in panels F and G), whereas other cells were squamous in shape, resembling type I cells (arrowheads) and positive for the type I cell marker OTS‐8 as previously reported (Vanderbilt et al. 2015). CEACAM6 staining was observed in some cuboidal cells and more extensively in flattened epithelial cells (arrowheads, panels e and g) where co‐localization with OTS‐8 (Fig. 5) and AQP5 (Fig. 7) were observed. There was some co‐localization of CEACAM6 with cytoplasmic EGFP of cuboidal cells (panel G, arrow and insets). However, flattened epithelial cells were primarily either EGFP^+^ or CEACAM6^+^, which is consistent with two separate precursor cell sources for new type I cells: type II cells and CEACAM6^+^ non‐type II cells.

**Figure 9:**
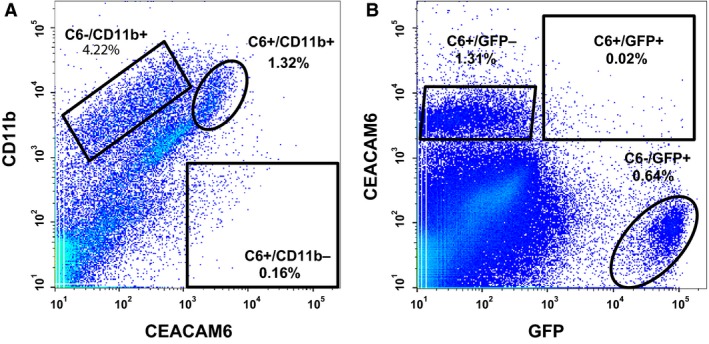
Representative scatter plots for sorting of lung cells. (A) Cells isolated from a bleomycin‐treated CEABAC mouse. In this 2‐way sort of live/single cells, 0.16% were CEACAM6^+^ and negative for CD11b, a lymphoid marker (box). 100,000 cells were recorded. B, Cells isolated from bleomycin‐treated CEABAC/CBG mice. A 2‐way sort provided populations of EGFP ^+^ cells that were negative (square) or positive (ellipse) for CEACAM6. 1,000,000 cells were recorded.

We performed cell sorting to further examine expression of CEACAM6 in alveolar epithelial cells after bleomycin injury. Lungs were instilled with elastase to preferentially release epithelial cells of alveoli and smaller airways, although some mesenchymal and larger airway cells are also isolated by this procedure. We first addressed the question of whether there are differences in number of CEACAM6^+^ cells with and without bleomycin injury of CEABAC mice. Because neutrophils (and other lymphoid cells) can express CEACAM6 on activation, we sorted for both CEACAM6 and CD11b, a marker of lymphoid cells. A representative cell sort for a bleomycin‐treated mouse is shown in Figure 9A and results are summarized in Table 2 (upper, CEABAC mice). Approximately 4–6% of sorted cells were positive for CD11b with no difference in abundance between control and bleomycin‐treated lungs. The number of CEACAM6^+^/CD11b^‐^ cells was low (<2/1000) with a 3.6‐fold increase after bleomycin treatment, consistent with the immunostaining results for injured areas.

**Table 2:** Fluorescence activated sorting of lung cells

	Bleomycin	Control	Fold change	*P*
CEABAC Mice				
CD11b^+^ (% of single cells)	5.9 ± 1.9	3.9 ± 1.8	1.5	NS
CEACAM6^+^/CD11b^−^ (% of single cells)	0.18 ± 0.10	0.05 ± 0.04	3.6	0.02
CEABAC/CBG Mice				
Total EGFP^+^ (% of single cells)	0.31 ± 0.25	0.31 ± 0.23	1.0	NS
CEACAM6^+^/EGFP^+^ (% of EGFP^+^ cells)	3.7 ± 1.3	1.6 ± 1.2	2.3	0.02

Data are mean ± SD for 100,000 recorded cells/sort in 5 experiments with CEABAC mice and 8 experiments with CEABAC/CBG mice.

In further cell sorting studies we used CEABAC/CBG mice to examine CEACAM6 expression specifically in type II cells (Fig. 9B and Table 2 CEABAC/CBG mice). Approximately 0.3% of mixed lung cells isolated from CEABAC/CBG mice were positive for EGFP with no significant difference between control and bleomycin animals. Of the EGFP^+^ cells, 1.6% were positive for CEACAM6 for control animals versus 3.7% after bleomycin injury, a significant 2.3‐fold difference. Thus, following lung injury there is a transient increase in CEACAM6^+^ type I cells (Fig. 5 and 7A) and CEACAM6^+^ type II cells (Table 2) but not for other differentiated cell types examined (Figure S1).

We examined cell cycle status of CEACAM6^+^ cells in experiments using intensity of propidium iodide staining as an index of DNA content. For CEACAM6^+^ cells sorted from bleomycin‐injured CEABAC/CBG mice, the percent of single cells in G2/M phases was 4.5 ± 2.7% and 15.7 ± 7.7% (mean ± SD, *n* = 4, *P* = 0.02) for EGFP^+^ and EGFP^‐^ cells, respectively; a similar finding of higher proliferation rate in non‐type II CEACAM6^+^ cells was found for control mice (4.2 ± 3.4% vs. 10.9 ± 3.2%, respectively, *P* < 0.05). Collectively, these results suggest that there is a small population of non‐type II cells expressing CEACAM6 that have a relatively high proliferative rate, consistent with recent findings in injured lungs of infants (Gonzales et al. 2015), however we cannot rule out the possibility that some cells could be arrested in G2 phase.

### Other lung injury models and CEACAM6

Instillation of bleomycin represents one (imperfect) model of infant bronchopulmonary dysplasia. In order to evaluate the generality of the CEACAM6 response to various types of lung injury, we used other injury models relevant to infant lung disease. We injured lungs with hyperoxia or LPS, insults that frequently occur in the newborn premature infant. We also examined mice null for SP‐D, which produces a chronic pulmonary metabolic disorder that is similar to inherited deficiency of SP‐C in infants and children.

**Figure 10:**
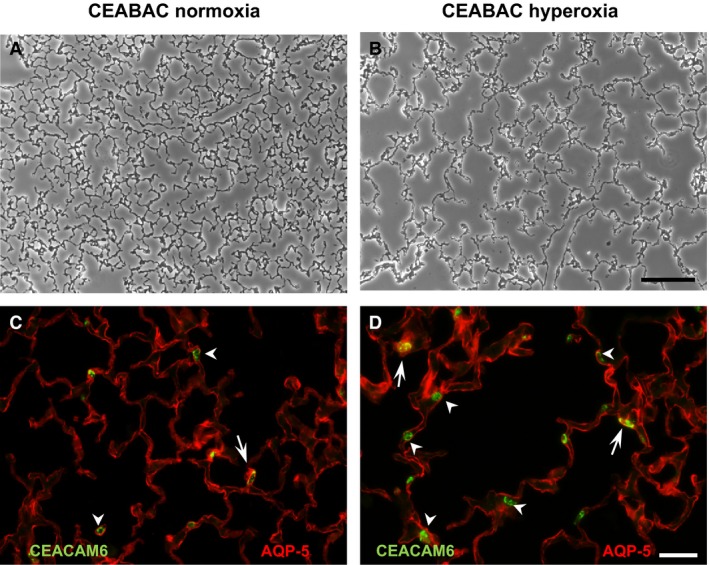
Lung structure and CEACAM6 expression in mice exposed to hyperoxia. Representative images of newborn CEABAC mice exposed to room air (A,C) or 80% oxygen (B,D) for 7 days. Note the simplified septal morphology and increased airspace size in mice exposed to hyperoxia (B vs. A, bar = 10 *μ*m). C versus D, CEACAM6 and AQP5 staining. In mice exposed to hyperoxia there was a 1.8‐fold increase in CEACAM6^+^ cell (green, arrows and arrowheads) compared to normoxic mice. There were similar numbers of cells that expressed both CEACAM6 and AQP5 (arrows) in each group. Bar = 40 *μ*m

We exposed neonatal mice to hyperoxia (80% for 7 days), which results in a generalized oxidative injury, and performed blinded counts of CEACAM6^+^. The number of CEACAM6^+^ alveolar epithelial cells were 1.8 ± 0.2 (mean ± SD, *P* < 0.05, *n* = 4) times greater in mice exposed to hyperoxia compared to normoxia; intensity of CEACAM6 staining was consistently greater in the hyperoxia samples (Fig. 10). As expected, alveolar development was impaired in the hyperoxia‐exposed mice as assessed by morphology (Fig. 10A vs. B) and by mean point counts (29.8 ± 2.6 vs. 38.6 ± 3.5 grid intersect points per 20X field, *P* = 0.02 hyperoxia vs. nomoxia).

One day after instillation of 500 *μ*g LPS to adult mice, large numbers of CEACAM6^+^ cells were observed within alveoli, reflecting an influx of activated, CD45^+^ (not shown) neutrophils (Figure S2A and phase contrast in part B). Because of the high lethality of this dose, subsequent experiments were performed with 50 *μ*g, which allowed survival of most animals for at least 2 weeks. At 10 days postinstillation, intra‐alveolar CEACAM6^+^ cells were no longer observed and some alveolar epithelial cells exhibited increased CEACAM6 signal (Figure S2C and D, arrows) at an intensity level above that observed for uninjured animals but less than seen after bleomycin; as in the bleomycin experiments, CEACAM6^+^ cells often had a flattened shape. By Western analysis, CEACAM6 content of lung homogenate was increased at 1–4 days (+58.7 ± 11.7%, *n* = 5, *P* = 0.001), reflecting CEACAM6 from neutrophils within airspaces; CEACAM6 in homogenate was not different from uninjured animals at day 10, consistent with clearance of neutrophils and modestly elevated expression of CEACAM6 in epithelial cells (data not shown).

We also assessed CEACAM6 expression levels in transgenic mice that were heterozygous for SP‐D and CEACAM6. SP‐D^−/−^ mice develop a chronic pulmonary inflammatory condition resulting in a metabolic dysfunction beginning at about 1 month that is characterized by increased alveolar surfactant, foamy macrophages, and type II cell hypertrophy with giant lamellar bodies (Botas et al. 1998). We examined the double transgenic animals at 3 months and found no apparent difference (compared to SP‐D^−/−^ mice) in lung morphology by light microscopy of plastic lung sections (Figure S3A vs. B). The CEACAM6 expression level by immunohistochemistry was not different in the double transgenic mice compared to uninjured CEABAC animals (*n* = 3 from each group). We conclude that the chronic changes caused by deficiency of SP‐D are not associated with stimulated CEACAM6 expression. Thus, CEACAM6 expression increases in lung alveolar epithelial cells, to varied degrees, with three different types of lung injury but not in a chronic metabolic dysfunction disorder.

## Discussion

CEACAM6 has several potential functions in the lung in vivo including clearance of microorganisms, stabilization of surfactant and anti‐apoptotic activity, properties that are consistent with a role in the response to lung injury. In this study we used the CEABAC transgenic mouse model to explore the cellular distribution and expression level of human CEACAM6 in normal and injured lung. We confirmed the original observations of Chan and Stanners (Chan and Stanners 2004) of pulmonary CEACAM6 expression. Moreover, we found that tissue levels were similar to those of normal newborn infants and that the protein was associated with alveolar surfactant, supporting the relevance of the model. By immunohistochemistry, CEACAM6^+^ cells were detected in some alveoli of normal lung, however staining rarely co‐localized with markers of either type I or type II cells. CEACAM6 was up‐regulated during the repair phase after bleomycin and hyperoxic lung injury, co‐localizing with markers of type I and type II cells but not other cell types. We speculate that CEACAM6 in this model is a marker of a lung progenitor cell population, which contributes to replenishing alveolar cells after injury of the epithelium, and that CEACAM6 promotes cellular repair via its anti‐apoptotic and adhesive properties.

There are several lines of evidence supporting the novel concept of a CEACAM6‐expressing progenitor cell in the lung. First, the proportion of detected CEACAM6^+^ cells in normal alveolar epithelium is low (<1%), consistent with a small subpopulation of cells. Second, the time course for increased number and signal intensity of CEACAM6^+^ cells after bleomycin coincides with the repair phase after injury as appropriate for repopulation of the epithelium. Third, cells with increased intensity of CEACAM6 staining are found only in injured areas of the lung, indicating localized regulation of the cellular response. Fourth, CEACAM6^+^ cells have an elevated mitosis rate, consistent with both the anti‐apoptotic activity of CEACAM6 and proliferation associated with cell replenishment. Finally, increased co‐expression of CEACAM6 with both type I and type II cell markers during the repair process, and loss of such co‐localization by 28 days after bleomycin, is the expected pattern for differentiation of CEACAM6^+^ cells into type I and type II cells of the renewed epithelium. Additional studies are required to test this proposal of progenitor status and to characterize the phenotype of both basal and proliferating CEACAM6‐expressing cells.

Our findings likely relate to the well‐established role of CEACAM6 (and CEACAM5) as human oncoproteins. As recently reviewed (Blumenthal et al. 2007; Beauchemin and Arabzadeh 2013), CEACAM6 is overexpressed in colonic hyperplastic polyps and early adenomas as one of the earliest molecular changes, and levels of the protein predict risk for metastases and poor survival outcome (Kuroki et al. 1999; Jantscheff et al. 2003). Overexpressing or silencing CEACAM6 in cultured colorectal cancer cell lines enhances and impedes invasion through the extracellular matrix, respectively. For breast cancer, CEACAM6 expression levels are associated with tamoxifen resistance and recurrence of disease, and silencing of CEACAM6 in cancer cells reversed endocrine dependence and anchorage independence, which promotes metastasis. In multiple myeloma, treatment with anti‐CEACAM6 mAbs, or siRNA to silence CEACAM6 reinstated T cell reactivity against malignant plasma cells. CEACAM6 is also a marker for pancreatic cancers and is being investigated as a therapeutic target; it is implicated in metastatic activity of pancreatic cancers via promotion of epithelial–mesenchymal transition (Chen et al. 2013). Finally, most lung adenocarcinomas express high levels of CEACAM6 protein, which are associated with poor clinical outcome (Blumenthal et al. 2007; Kobayashi et al. 2012; Lee et al. 2015). Using lung‐derived A549 cells, Han et al. (Han et al. 2014) demonstrated a role for CEACAM6 in tumor growth in vivo and proliferation, migration, and invasion properties in vitro. Collectively, these observations in both patients and cell lines establish a functional role for CEACAM6 in early events of tumorgenesis and in metastatic behavior of cancers of the lung and other tissues, which are mediated by effects of dysregulated CEACAM6 expression on cell proliferation, adhesion, and migration. We propose that these functions are also involved in repair of injured lung epithelium in CEABAC mice.

CEABAC mice express CEACAM6 and CEACAM5 at similar mRNA levels, however we could not confirm that the two proteins are co‐expressed in the same cell because of the unavailability of an anti‐CEACAM5 antibody that does not cross‐react with CEACAM6. The transgenic mice also express CEACAMs 3 and 7 mRNAs, albeit at low levels, but protein expression was not detected in previous studies (Chan and Stanners 2004). We could not determine whether functions of CEACAMs 5 and 6 in lung cells are similar, as observed in human cells, nor can we rule out that their expression levels, cell specificity or functions might be altered by expression in mouse versus human lung cells. However, the relevance of CEACAM6 expression results in the mouse for human lung is supported by the recent findings for cellular distribution and increased content and staining intensity of CEACAM6 in injured lungs of human infants (Gonzales et al. 2015).

Under normal conditions, turnover of lung cells is relatively slow compared to organs such as skin and intestine, with an estimate of 28–35 days for alveolar cells of the mouse (Bowden et al. 1968; Kauffman 1980). Activation of repair mechanisms after injury from agents such as hyperoxia, ozone, NO2 and bleomycin involves proliferation of progenitor cells to repopulate the alveolar epithelium, however the identification of these stem cells remains uncertain. Early data with tritiated thymidine pulse labeling indicated that type II cells can give rise to type I cells after NO2 exposure (Evans et al. 1975), and more recent lineage tracing studies have confirmed this event under both normal and post‐bleomycin conditions (Wansleeben et al. 2013; Desai et al. 2014). Our current and previous (Vanderbilt et al. 2015) findings with CBG mice support a role for type II cells in differentiation of type I cells after lung injury. However, there is evidence for other candidate progenitor cells. Chapman et al. (Chapman et al. 2011) reported regenerative potential of alveolar cells expressing the laminin receptor a6b4, and negative for SFTPC, for type II cells after bleomycin injury. There is also evidence in mice for a population of bronchoalveolar stem cells (BASCs), which co‐express Scgb1a1 and sftpc, that give rise to both bronchiolar and alveolar cells in culture, repopulate bronchioles after naphthalene injury and contribute to type II cells after bleomycin (Kim et al. 2005); interestingly, an increase in BASCs was observed at 14 days, but not at 7 or 28 days after bleomycin, which is similar to the time course for increased CEACAM6 expression. In our studies with CEABAC/CBG mice, we found that flattened epithelial cells in bleomycin‐injured areas were primarily either EGFP^+^/CEACAM6^−^ (i.e., arising from type II cells) or EGFP^−^/CEACAM6^+^, indicating a non‐type II cell origin. These findings are consistent with differentiation of type II cells into type I cells, as demonstrated by others, and with the existence of a non‐type II cell progenitor population expressing CEACAM6. We cannot rule‐out the possibility that CEACAM6^+^ progenitor cells first differentiate into type II cells and subsequently into type I cells.

The stimuli for increased expression of CEACAM6 after lung injury are not known. In human fetal lung, CEACAM6 is up‐regulated by glucocorticoids plus cAMP (Kolla et al. 2009), however it seems unlikely that these agents are involved locally in injured tissue areas. The oxidative and nitrative stress associated with bleomycin evokes a cytokine/chemokine response (Cavarra et al. 2004), which could stimulate CEACAM6 expression. It has been proposed that substances released from damaged type I cells, including EGFR ligands, could stimulate proliferation and differentiation of progenitor cells (Desai et al. 2014); this response could involve CEACAM6 up‐regulation. It is reported that CEACAM6 is regulated by HIF1A (Mimouna et al. 2011, 2014), which could be activated in hypoxic, injured areas of the lung after bleomycin. There is evidence that CEACAM6 expression is influenced by mir‐29a (Han et al. 2014), suggesting the possibility of a secondary regulatory process for CEACAM6 gene expression. Additional studies are required to address the mechanism of delayed CEACAM6 up‐regulation during the repair phase after injury.

The findings of increased CEACAM6^+^ cells in alveoli of bleomycin‐treated CEABAC mice likely have relevance for lung injury in the human. In expression profiling experiments with postmortem lung of infants, CEACAM6 transcript was increased in lungs of infants with bronchopulmonary dysplasia compared to control tissue (Bhattacharya et al. 2012). In immunohistochemical studies, we have detected a strong CEACAM6 expression, including co‐localization with markers of both type I and type II cells, in hyperplastic alveolar cells of lungs from infants with a variety of chronic lung diseases (Chapin et al. 2012); staining for both type I and type II cell markers in a single cell, which is indicative of a transitional alveolar cell, is not found in normal infant (unpublished) or adult (Vanderbilt et al. 2015) lung. Although it is not possible to study the time course of CEACAM6 expression in postmortem specimens of human lung, it is likely that lung disease evokes proliferation of CEACAM6^+^ cells in injured alveoli with subsequent epithelial repair or continuing cell proliferation in more severe, chronic cases. Thus, CEACAM6 may also be a marker of alveolar progenitor cells in human lung and may participate in proliferation, migration and differentiation of these cells after injury. CEACAM6 may have a similar role during normal lung development in utero; previously we demonstrated increasing CEACAM6 expression in fetal lung during the third trimester, regulation by TTF‐1, a transcription factor that regulates numerous key genes of type II cells, and co‐localization of CEACAM6 in fetal lung with markers of both type I and II cells (Kolla et al. 2009; Gonzales et al. 2015). Future studies will need to examine CEACAM6 expression in lungs of adult patients with diseases involving epithelial damage and repair. Moreover, it is possible that levels of CEACAM6 in lung lining fluid and/or the circulation will reflect the severity of lung disease in infants (and adults) as found for specific cancers (Beauchemin et al. 2015).

There are some apparent differences in lung cellular distribution of CEACAM6 between adult humans and 3‐month old CEABAC mice. Whereas airway epithelium in both species demonstrates strong staining, we previously detected CEACAM6 immunoreactivity in most or all type I cells and in ~50% of type II cells of adult human lung; the signal was apical for type II cells and less strong and punctate for type I cells (Chapin et al. 2012). By contrast, CEACAM6 was not detected in most alveolar epithelial cells and rarely co‐localized with either type I or II cells in CEABAC mouse lungs under basal conditions. This apparent difference for CEACAM6 cellular distribution for adult mouse versus human lung could represent stronger gene expression in humans than mice, which were heterozygous, and/or detection limits with immunohistochemistry. Nevertheless, CEABAC mice appear to be a relevant model for human infants with lung disease.

In conclusion, there are several putative biological roles for the multifunctional CEACAM6 protein in the lung. In other tissues, and in in vitro studies, CEACAM6 binds a variety of gram‐negative bacteria and promotes internalization and phagocytosis (Chen et al. 1997); it is reasonable to assume that the protein has a similar role in lung innate immune defense, however this has not been directly studied. CEACAM6 is tightly associated with surfactant isolated from lung fluid of premature infants (Chapin et al. 2012), and this observation was confirmed with CEABAC animals. Previously, we reported that the presence of CEACAM6 protected surfactant function from the inhibitory effects of serum proteins in vitro (Kolla et al. 2009), and the same effect could occur in vivo, particularly during lung injury with increased alveolar protein content. A third property of CEACAM6 is its anti‐apoptotic activity, which has been demonstrated in transformed cell lines (Ilantzis et al. 1997; Ordonez et al. 2000) and in cultured human fetal lung cells (Kolla et al. 2009). CEACAM6 also mediates cell–cell adhesion, which is perturbed in lung adenocarcinomas. Other than the modest effect on weight loss, our studies indicate that the presence of human CEACAMs in lung of the mouse, which does not have the CEACAM5/6 genes, does not affect the severity of injury by bleomycin. Rather, we demonstrate that increased expression of CEACAM6 occurs in the repair phase after lung injury, and we speculate that CEACAM6 is a biomarker of a population of alveolar progenitor cells and participates in epithelial replenishment by virtue of its anti‐apoptotic and adhesive activities.

## Conflict of Interest

None declared.

## Supporting information




**Figure S1.** Immunostaining for CEACAM6 and non‐epithelial cell markers in bleomycin‐treated CEABAC mice. Representative sections are shown for *α*‐smooth muscle actin (*α*‐SMA, A–C), vimentin, marker for fibroblasts (D–F), CD45, marker for lymphoid cells (G–I) and calcitonin gene related peptide, marker for neuroendocrine cells (CGRP (CALCA), J–K). No co‐localization with CEACAM6 was observed for any of these cell markers.

**Figure S2.** Representative immunohistochemistry for CEACAM6 after instillation of LPS. CEABAC mice received 20 *μ*g/g LPS (A, B) or 2 *μ*g/g (C, D) and lung sections were immunostained for CEACAM6 (green) and OTS‐8 (red) at 2 days (A) and 10 days (C); corresponding phase contrast images are shown in B and D. Note intra‐alveolar CEACAM6^+^ cells at 2 days (green, arrows) and positive epithelial staining (arrows) at 10 days. Bar = 20 *μ*m.

**Figure S3.** Studies of CEACAM6 expression in SP‐D‐null mice. Panel A, Lung morphology in plastic sections of SP‐D^−/−^ (A) and SP‐D^+/−^/CEABAC mouse (B) at 3 months of age. Both animals demonstrate increased alveolar surfactant, foamy macrophages, and type II cell hypertrophy with giant lamellar bodies, indicating no effect of human CEACAM6 expression on progression of lung disease in this model. Panel B, Representative immunostaining for CEACAM6 and AQP5 at 3 months of age. In SP‐D^−/−^ mice (control, A) no CEACAM6 signal is seen as expected. There is a similar low level of CEACAM6 staining In the CEABAC mouse (B) and the SP‐D^+/−^/CEABAC mouse (C).

**Figure S4.** Total protein on Western blots by amido black staining. (A) Staining for blot representative of CEACAM6 immunostaining in Figure 1A. (B) Staining for blot representative of CEACAM6 immunostaining in Figure 4A. Total protein loading in *μ*g is shown for each lane at the top of the blot and is reflected in the intensity of staining for major protein bands.
