# Supplementary figures and images for: Expression of human carcinoembryonic antigen‐related cell adhesion molecule 6 and alveolar progenitor cells in normal and injured lungs of transgenic mice
Source: Physiol Rep. 2015 Dec 23;3(12):e12657. doi: 10.14814/phy2.12657 (PMC4760449; doi:10.14814/phy2.12657)

Supplemental Figure 1

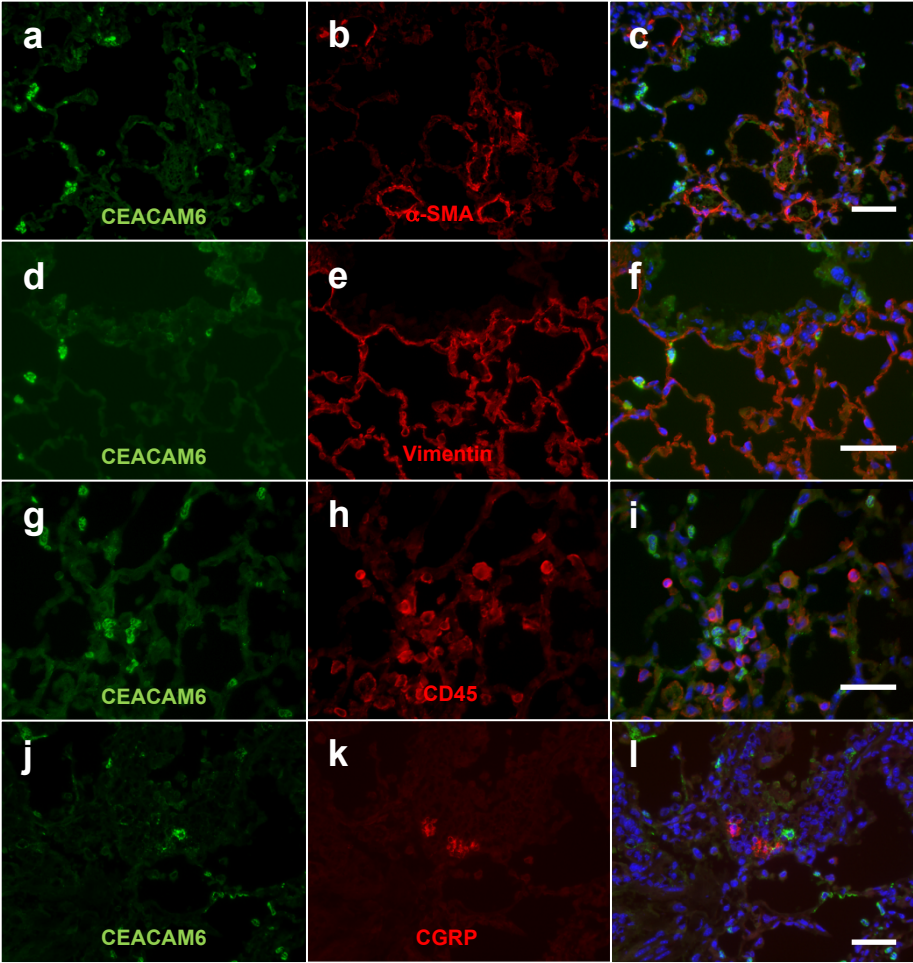

Supplement: Supplementary file 1 — Figure S1. Immunostaining for CEACAM6 and non‐epithelial cell markers in bleomycin‐treated CEABAC mice. Representative sections are shown for α‐smooth muscle actin (α‐SMA, A–C), vimentin, marker for fibroblasts (D–F), CD45, marker for lymphoid cells (G–I) and calcitonin gene related peptide, marker for neuroendocrine cells (CGRP (CALCA), J–K). No co‐localization with CEACAM6 was observed for any of these cell markers. [file PHY2-3-e12657-s001.pdf]

Supplemental Figure 2

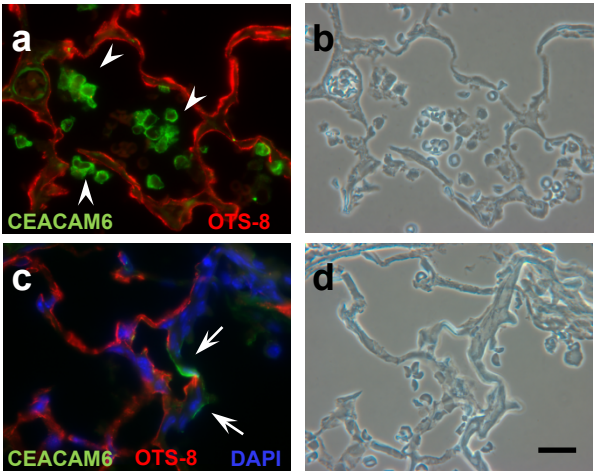

Supplement: Supplementary file 2 — Figure S2. Representative immunohistochemistry for CEACAM6 after instillation of LPS. CEABAC mice received 20 μg/g LPS (A, B) or 2 μg/g (C, D) and lung sections were immunostained for CEACAM6 (green) and OTS‐8 (red) at 2 days (A) and 10 days (C); corresponding phase contrast images are shown in B and D. Note intra‐alveolar CEACAM6+ cells at 2 days (green, arrows) and positive epithelial staining (arrows) at 10 days. Bar = 20 μm. [file PHY2-3-e12657-s002.pdf]

Supplemental Figure 3

**A**

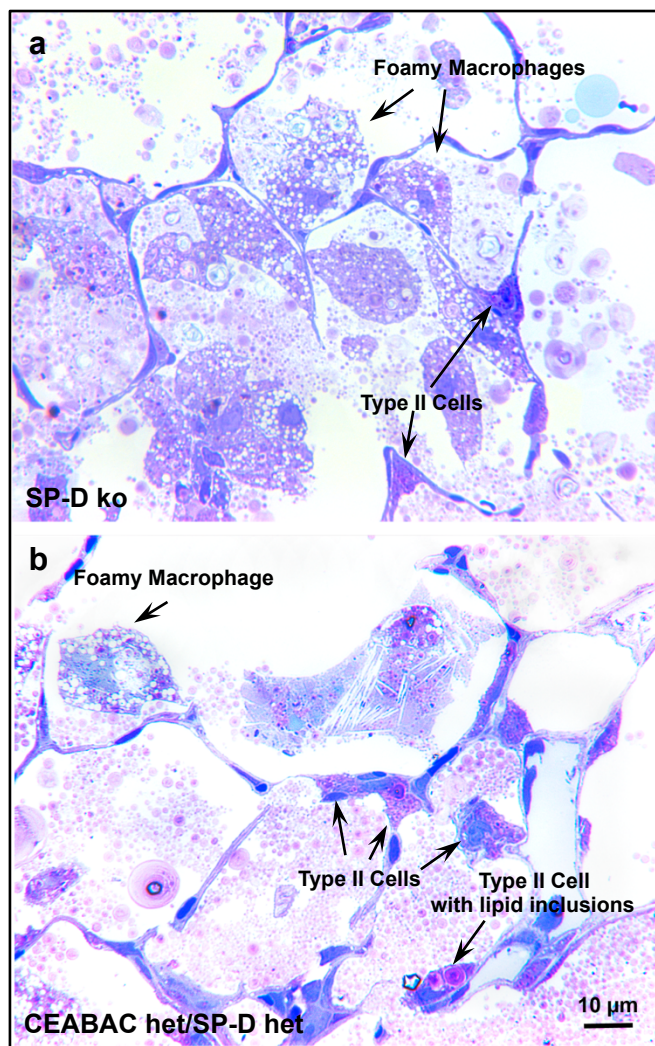

**B**

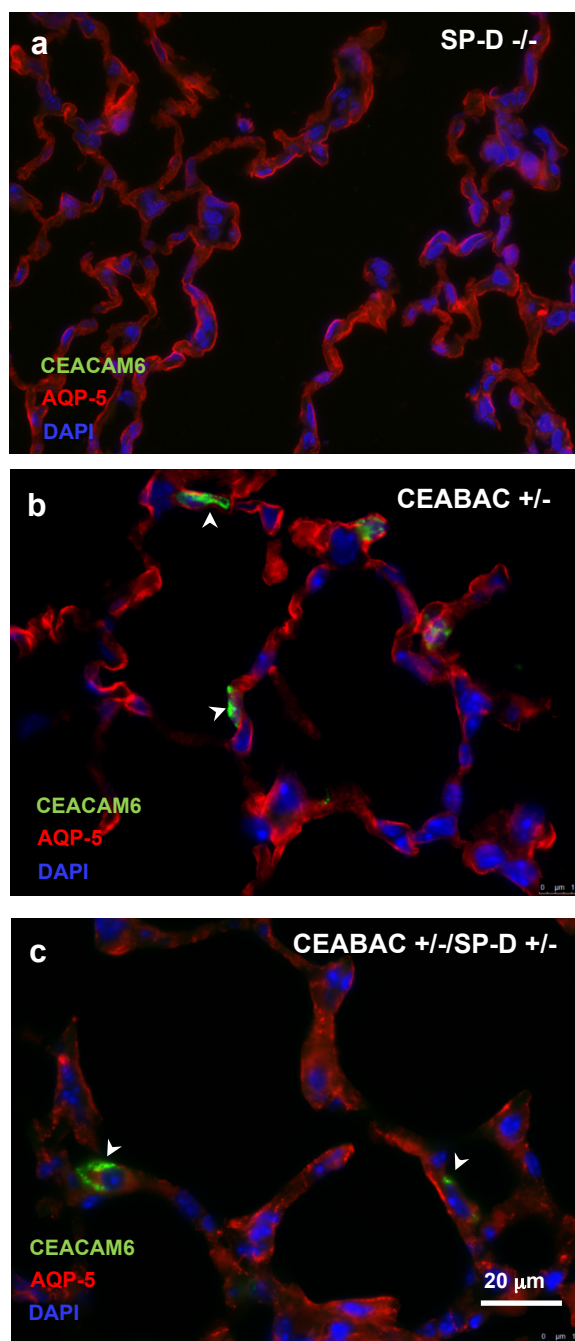

Supplement: Supplementary file 3 — Figure S3. Studies of CEACAM6 expression in SP‐D‐null mice. Panel A, Lung morphology in plastic sections of SP‐D−/− (A) and SP‐D+/−/CEABAC mouse (B) at 3 months of age. Both animals demonstrate increased alveolar surfactant, foamy macrophages, and type II cell hypertrophy with giant lamellar bodies, indicating no effect of human CEACAM6 expression on progression of lung disease in this model. Panel B, Representative immunostaining for CEACAM6 and AQP5 at 3 months of age. In SP‐D−/− mice (control, A) no CEACAM6 signal is seen as expected. There is a similar low level of CEACAM6 staining In the CEABAC mouse (B) and the SP‐D+/−/CEABAC mouse (C). [file PHY2-3-e12657-s003.pdf]

Supplemental Figure 4

Whole Lung Homogenate: Amido Black

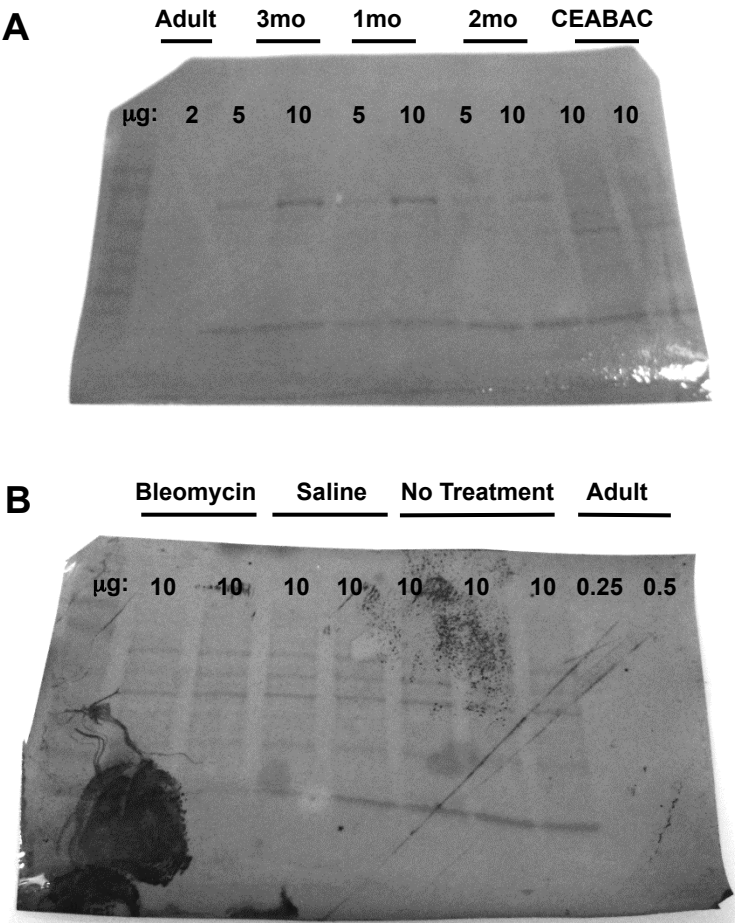

Supplement: Supplementary file 4 — Figure S4. Total protein on Western blots by amido black staining. (A) Staining for blot representative of CEACAM6 immunostaining in Figure 1A. (B) Staining for blot representative of CEACAM6 immunostaining in Figure 4A. Total protein loading in μg is shown for each lane at the top of the blot and is reflected in the intensity of staining for major protein bands. [file PHY2-3-e12657-s004.pdf]
